# Glutamine administration in sepsis: enteral, parenteral or both? Experimental study in swine

**DOI:** 10.1186/cc14484

**Published:** 2015-03-16

**Authors:** G Stavrou, E Filidou, K Arvanitidis, K Fotiadis, V Grosomanidis, A Ioannidis, G Tsaousi, A Michalopoulos, G Kolios, K Kotzampassi

**Affiliations:** 1 Aristotle University of Thessaloniki, Greece; 2 Democritus University of Thrace, Alexandroupolis, Greece

## Introduction

Glutamine (GLN) is recommended in the critically ill for i.v. administration but enteral use is not quite clarified. We decided to measure GLN plasma levels in healthy and septic swine after GLN given by i.v., enteral or both routes, over a 3-hour period.

## Methods

Sepsis was induced by *E. coli *LPS. GLN was infused: i.v. through the femoral vein (0.5 g/kg); enterally (E) through jejunostomy (0.5 g/kg); and i.v. + E. Blood was drawn continuously from the femoral artery and the portal vein for GLN plasma levels in systemic-S and portal-P circulation.

## Results

**Figure 1:**
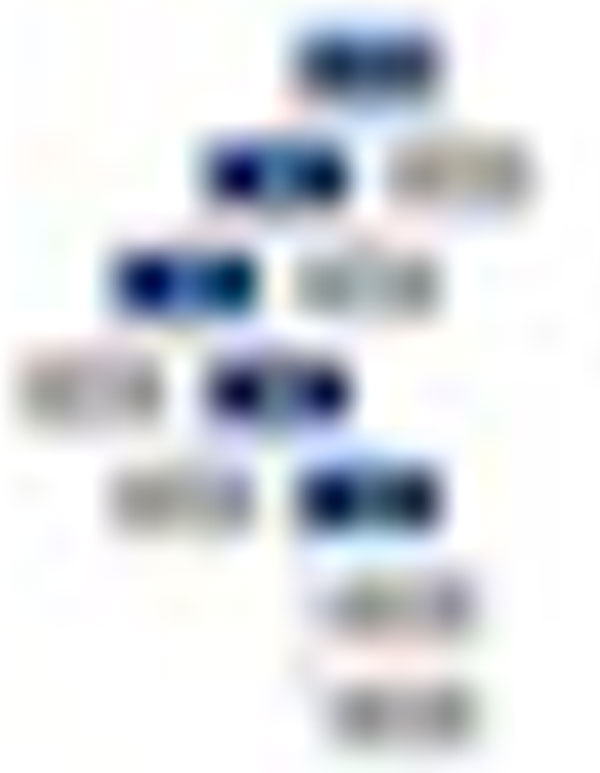


**Figure 2:**
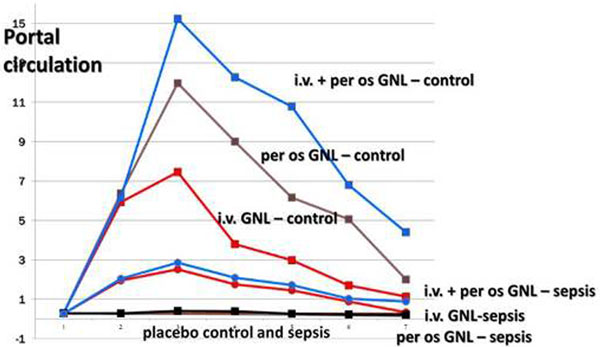


In healthy swine, GLN levels remained stable, both in S and P; i.v. infusion, and even more i.v. + E significantly increased GLN in the S circulation (*P *< 0.001), whereas E infusion failed to do so (*P *= 0.4). On the contrary, GLN P levels were significantly increased after i.v. + E infusion, as well as after E infusion (*P *< 0.001) and to a lesser extent, after i.v. (*P *< 0.001). In sepsis, both S and P GLN levels decreased significantly. As previously, i.v. (*P *= 0.001) and even more i.v. + E (*P *< 0.001) infusion significantly increased S GLN levels, while E infusion failed to have any effect. In the P vein, both i.v. (*P *= 0.02) and i.v. + E (*P *< 0.001) GLN increased significantly, whereas the E had no effect (*P *= 0.08). See Figures 1 and 2.

## Conclusion

In our experimental early sepsis model, a combination of E and i.v. GLN seems to be the most appropriate; this Results in high GLN levels for the functional needs, including those of the gut mucosa.
